# Dietary Fluoride Exposure During Early Childhood and Its Association with Dental Fluorosis in a Sample of Mexican Adolescents

**DOI:** 10.3390/ijerph22050689

**Published:** 2025-04-26

**Authors:** Gina A. Castiblanco-Rubio, Emily C. Hector, Jose Urena-Cirett, Alejandra Cantoral, Howard Hu, Karen E. Peterson, Martha M. Tellez-Rojo, E. Angeles Martinez-Mier

**Affiliations:** 1 Department of Dental Public Health and Dental Informatics, Indiana University School of Dentistry, Indianapolis, IN 46203, USA; ginacast@iu.edu; 2 Department of Statistics, North Carolina State University, Raleigh, NC 27695, USA; ehector@ncsu.edu; 3 Faculty of Dentistry, Universidad La Salle Mexico, Mexico City 01376, Mexico; joseluis.urena@lasalle.mx; 4 Health Department, Universidad Iberoamericana, Mexico City 01376, Mexico; alejandra.cantoral@ibero.mx; 5 Department of Preventive Medicine, Keck School of Medicine, University of Southern California, Los Angeles, CA 90033, USA; howard.hu@med.usc.edu; 6 Department of Nutritional Sciences, School of Public Health, University of Michigan, Ann Arbor, MI 48109, USA; karenep@umich.edu; 7 Center for Nutrition and Health Research, National Institute of Public Health, Cuernavaca 62100, Morelos, Mexico; mmtellez@insp.mx

**Keywords:** fluorides, trace elements, dental fluorosis, environmental biomarkers, dietary exposure, preschool child

## Abstract

Dental fluorosis indicates past fluoride intake. People living in Mexico City are exposed to fluoridated salt, which contributes significantly to fluoride intake. This study aimed to (1) estimate fluoride intake during early childhood and fluorosis prevalence in permanent teeth in adolescence and (2) identify intake windows associated with higher fluorosis scores in upper central incisors (UCIs). We included 432 participants from the ELEMENT project (Early-Life Exposures in Mexico to Environmental Toxicants), with data on fluoride intake at ages 1–5 and fluorosis (TFI) at adolescence. Median intakes ranged from 0.56 at age 1 to 1.14 mg/day at age 5, exceeding recommendations. All adolescents had some level of fluorosis, predominantly mild (62% with TFI 2). For every 0.1 mg of daily fluoride intake at age 1, the odds of higher TFI in UCIs were 1.08 [95% CI: 1.00–1.17]. At age 2, the odds were marginally significant [OR: 1.07; 95% CI: 1.00–1.16]. In conclusion, for participants of ELEMENT: (1) fluoride intake during early childhood exceeded recommendations and the prevalence of mild fluorosis in adolescence was high, and (2) fluorosis in UCIs was associated with dietary exposure during the first two years of life and may be used in future ELEMENT studies as exposure biomarkers.

## 1. Introduction

Dental fluorosis occurs when the mineralization process of dental enamel is disrupted due to prolonged exposure to fluoride during tooth development [1]. Although environmental factors and genetics play a role in the pathogenesis of dental fluorosis [2,3], there is consensus that its occurrence is closely linked to total fluoride intake levels [4]. The changes that result from the disturbed mineralization process can be clinically assessed in the enamel of the teeth after their eruption, and classified with different indices as mild, moderate, or severe [5,6]. Mild dental fluorosis is typically of no concern, but severe cases can negatively impact dental and oral health [1]. Furthermore, moderate to severe dental fluorosis serves as an endpoint of adverse health effects in environmental risk assessment [7] and for establishing dietary reference intakes of fluoride. The U.S Institute of Medicine recommends an Adequate Intake (AI) level of fluoride to help prevent dental caries and reduce the occurrence of moderate dental fluorosis. The recommended intake starts at 0.01 mg per day for infants and increases to 1 mg per day at age 5 [8].

Biologically, dental fluorosis can be equated to a hard tissue “scar” that reflects environmental fluoride exposure during specific periods in childhood (0–8 yrs), when most permanent teeth develop their crowns [9]. Permanent teeth develop their crowns sequentially by tooth groups, starting with the upper central incisors around the 4th postnatal month. By 5 years of age, most of the crowns are nearly complete for most permanent teeth, and around age 8, most teeth have fully mineralized their crowns [10]. The eruption of these teeth starts at around 6 years of age, and the permanent dentition (except for third molars or “wisdom teeth”) will be completely erupted by age 13. For this reason, the early teenage years are a target for the assessment of dental fluorosis in the permanent dentition, and for the monitoring of fluoride intake over time at the population level. Around the 1940s, pioneering epidemiological research by H.T Dean suggested that a prevalence of mild fluorosis of 10% could be used as a threshold for fluorosis to be recognized as a public health concern [11]. Since then, changes in the occurrence and severity of dental fluorosis across cohorts of adolescents have served as an indicator of changes in overall fluoride exposure over time. Data on fluorosis prevalence in the US across cohorts of adolescents (1999 to 2004 and 1986 to 1987) have suggested an increase in fluoride intake [12]. Consequently, new potential adverse health effects associated with current levels of exposure are being investigated [13].

Aside from population-level surveillance of early childhood fluoride exposure over time, dental fluorosis may serve as a biomarker of fluoride exposure at the individual level when assessed in specific groups of teeth [14]. Although critical windows of fluoride exposure for most teeth overlap, a body of research on fluoride intake and dental fluorosis has identified ages 0–3 as the most important ones for teeth that appear early in the oral cavity (e.g., upper central incisors and first molars) [4], and ages 2–8 for teeth appearing later (canines, first and second premolars, and second molars) [15]. The upper central incisors are the preferred tooth group to use as biomarkers, due to their early eruption in the oral cavity, ease of access for clinical assessment, and a body of evidence on critical windows of exposure, estimated between 0 and 3 years of age [4]. However, some authors have acknowledged that the effects of fluoride intake during any critical window of exposure are confounded by fluoride intake at other ages [16] and the identification of the maximum susceptibility window of a specific tooth group should control for fluoride intake at all ages of exposure during tooth development.

This study aimed to achieve the following: (1) assess the dietary intake of fluoride during early childhood among a sample of participants of the Early Life Exposures in Mexico to ENvironmental Toxicants (ELEMENT) project [17] and determine the prevalence and severity of dental fluorosis at adolescence; and (2) examine whether the severity scores of fluorosis in the upper central incisors reflect dietary fluoride intake during early childhood in this cohort. We hypothesized that the dietary intake of fluoride does not differ from recommendations and that the dietary intake of fluoride estimated at ages 1, 2, 3, 4 and/or 5, is associated with a one-score change in severity in dental fluorosis in the upper central incisors.

## 2. Materials and Methods

### 2.1. Analytic Sample and Setting

**Figure 1:**
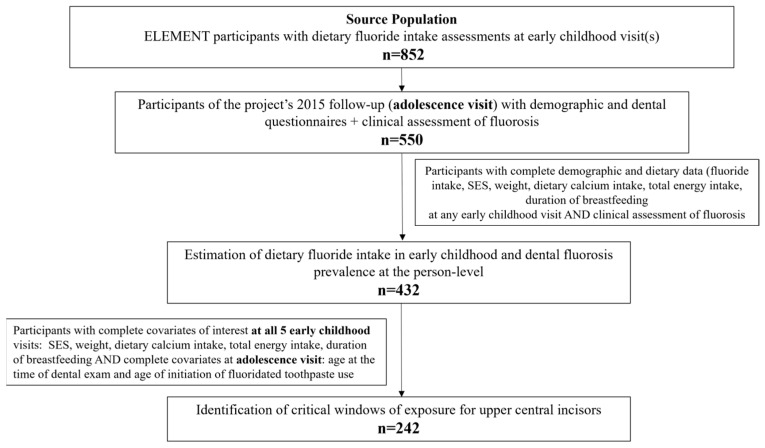
Flow chart of source population and analytic sample, drawn from the Early Life Exposures in Mexico to Environmental Toxicants (ELEMENT) project.

The source population was adolescents participating in the ELEMENT project, which has been described previously [17]. ELEMENT comprises three mother–child pregnancy and birth cohorts initiated in the 1990s to study early life exposures and health outcomes in Mexico City. The project has a dedicated research facility next to Mexico City’s ABC Medical Center, and participants were invited for follow-up visits during pregnancy (1997–2005), the child’s first five years of life (between 1998 and 2010), and adolescence (2015). Details and demographics of the entire project can be found in the ELEMENT project’s profile publication [17]. At each study visit, participant mothers and their children were interviewed by a trained nurse, who performed anthropometry (weight and height) using calibrated instruments and applied a general demographic questionnaire (that included information on breastfeeding) and a validated Food Frequency Questionnaire (FFQ). During the early childhood period, mothers answered questions regarding socioeconomic status. This questionnaire was created by the “Asociación Mexicana de Agencias de Investigación de Mercados y Opinión Pública” (Mexican Association of Marketing Research and Public Opinion Agencies) to evaluate household resources. The index consists of 7 categories (A, B, C+, C, D+, D, E) with “A, B” being the highest, to “E” the lowest. The adolescence follow-up visit included a clinical assessment of dental fluorosis and a dental health questionnaire (n = 550). The dental questionnaire included a question on the age of initiation of the use of fluoridated toothpaste. Only adolescents who had available the sociodemographic, anthropometric, and dietary data of interest at any childhood visits and clinical examination of dental fluorosis at adolescence were included to estimate dietary fluoride intake and dental fluorosis prevalence (n = 432). On the other hand, in the models to identify critical windows of exposure for the upper central incisors, only those with complete covariates of interest at all 5 early childhood visits (SES, weight, dietary calcium intake, total energy intake, duration of breastfeeding) and complete covariates at the adolescence visit (age at the time of dental exam and age of initiation of fluoridated toothpaste use), were included (n = 242) (Figure 1). The research protocols were approved by the Ethics and Research Committees of all participating institutions (National Institute of Public Health, the University of Michigan School of Public Health and Indiana University School of Dentistry, IRB# 137-6312, 100209 and 105-6134).

### 2.2. Estimation of Dietary Intake of Fluoride and Calcium in Early Childhood

The crowns of the upper central incisors begin their development around 5 postnatal months and are completely formed at around age 5 [9]. Therefore, we retrieved the dietary variables of interest (fluoride and calcium) from the FFQ for the first 5 years of life. We were interested in estimating calcium intake due to its role in decreasing the intestinal absorption of fluoride, which may confound the relationship between levels of intake and dental fluorosis [2]. Since 1981, a salt fluoridation program has been in place in multiple regions in Mexico (including Mexico City) to deliver fluoride for caries prevention at a concentration of 250 ppm of F/kg [18]. Salt fluoridation is mandatory in the region, and a subanalysis of fluoride content in samples of salt collected from a subset of ELEMENT households confirmed that almost all the families use fluoridated salt. Consequently, we worked under the assumption that the primary sources of dietary fluoride intake consist of foods containing intrinsic fluoride content or cooked with fluoridated salt. Our study evaluated dietary fluoride intake (mg F/day) using a semiquantitative questionnaire comprising 104 items. This questionnaire was adapted from the Willett semi-quantitative FFQ [19] to include foods commonly consumed in the 1983 Dietary Survey of the Mexican National Institute of Nutrition 20 [20] and validated to estimate dietary intake over the last three months in women of childbearing age living in Mexico City [19]. Mean ages of participants at early childhood visits were (in months): 11.8 ± 0.4 at year 1; 23.8 ± 0.4 at year 2; 35.6 ± 2.3 at year 3; 47.9 ± 0.8 at year 4, and 60.2 ± 1.0 at year 5 (Table 1) and the questionnaire was applied to the caregiver (usually the mother participating in the ELEMENT project). The FFQ was neither specifically designed nor validated for estimating fluoride. Since fluoride is not included in the INSP nutrient composition database, a specific fluoride database was created for the ELEMENT project by analyzing the fluoride content of typical foods in the Mexican diet, with details published elsewhere [21]. Although water is not expected to be the main source of dietary fluoride intake in Mexico City (but foods with intrinsic fluoride content or those cooked with fluoridated salt), our fluoride database included estimations on the levels of fluoride [mean ± SD] in both tap water [0.16 ± 0.13 ppm] and different brands of bottled water [0.14 ± 0.09]. To minimize sources of external variability, foods that are consumed cooked (meats, rice, pasta, and legumes) were boiled in the laboratory using bottled water (Evian®, USA) with negligible amounts of fluoride (<0.01 mg/L) and no added salt. Others, such as street foods were analyzed as purchased and no extra salt was added [21]. Therefore, fluoride intake levels from the FFQ are likely to be underestimations. Estimates of dietary fluoride and calcium intake (mg/day) were computed using software developed at the National Institute of Public Health (INSP). The software uses the reported frequencies and portion sizes from the FFQ, the INSP-compiled nutrient composition database, and the ELEMENT project’s fluoride database. The estimates derived from the FFQ include fluoride from beverages, tap, and bottled water, and fluoride from salt added to street foods during the cooking process [21], but not the amount of salt added to meals during home cooking or at the table.

### 2.3. Clinical Assessment of Dental Fluorosis

ELEMENT participants were eligible if (1) they were born to a mother who participated in the original cohorts, and (2) were followed during the first five years of age (n = 886). Details on the cohort’s original recruitment, general demographics, and follow-ups are provided elsewhere [17]. Following an invitation to participate and an information session by a study representative, 550 study participants provided assent to the clinical examination, and their parents/caregivers signed an informed consent form. At the time of examination, children were between 11 and 17 years old (mean age: 15 ± 2 years); inclusion criteria for the dental exam were to be in good general health, and those undergoing orthodontic treatment were excluded from participation (n = 48). Prior to their dental examination, the study’s participants were asked to thoroughly brush their teeth using water and a regular toothbrush to eliminate dental plaque from the surfaces. The dental exams were performed by an experienced pediatric dentist (JLU) trained in Thysltrup and Fejerskov Index (TFI) and calibrated against a senior examiner (EAMM) over the course of two days. On day one, a theoretical session and discussion of cases with patients was led by EAMM. On day two, both the senior and trainee examiners performed exams on 33 patients, with the trainee reaching “almost perfect” agreement [22] with the senior examiner (weighted kappa = 0.93). Clinical exams were performed under white light in a portable chair with No. 5 front surface mirrors and cotton rolls. The pediatric dentist (JLU) was also trained and experienced in the differential diagnosis of other developmental enamel defects (DDE), including MIH with EAPD criteria and diffuse/demarcated opacities with Russel’s criteria, which were judged at the chairside and assigned a specific code. Then, JLU assigned a TFI score ranging from 0 to 9 to each tooth’s buccal, lingual, and occlusal surfaces. A trained dental assistant recorded the data on a paper form that was later entered into a database using FoxPro System 2.6 (Fox Software Microsoft, Perrysburg, OH, USA).

### 2.4. Dental Fluorosis Variable

To define a single score at the tooth level, the highest TFI score by surface was considered. Then, the highest score among the two teeth belonging to a group (e.g., upper incisors, upper first molars) was assigned to define the score of that tooth group. To define the severity distribution at the person level, we used the highest tooth group score reported for each individual, considering all their erupted permanent teeth. For the assessment of associations between fluoride intake and dental fluorosis, in statistical models, we included only TFI scores for the upper central incisors.

### 2.5. Data Analyses

Exploratory statistics to describe the sample and variables of interest were performed. We checked the distribution of dietary fluoride intake and covariates of interest at each time point using summary measures (means, medians, and measures of dispersion, as appropriate), plots, and histograms. The comparison among median estimated dietary fluoride intake and AI recommendations was performed using Wilcoxon signed-rank test. The distribution of TFI scores was summarized using frequency distributions at the individual (person) level, and by tooth group. Cumulative logit link regression models were used to estimate the association between dietary fluoride intake (once per year, at the end of each one of the yearly windows from 1 to 5 years of age) and ordinal TFI scores in the upper central incisors, adjusting for the following covariates assessed in the early childhood visits: birth cohort, socioeconomic status (SES), duration of breastfeeding (months), dietary calcium intake (mg/day), total dietary intake (kcal/day) and weight (kg); and the following assessed in the adolescence visit: age at the time of dental exam and age of initiation of fluoridated toothpaste use (yrs). To prevent collinearity among predictors with measurements for each time point at early childhood visits (calcium intake, weight, and total energy intake), summaries were obtained using four principal components for each variable.

During tooth development, each window of exposure may contribute differently to the pathogenesis of fluorosis. To account for the confounding effect of overlapping exposure windows, we generated a fixed effects model that included crude dietary fluoride intake (in mg/day) at each age (1 to 5) both as explanatory variables and covariates and TFI scores in the upper central incisors as outcomes. Given that the median fluoride intake ranged from 0.5 to 1.1 mg/day across early childhood, for a meaningful interpretation, this variable is reported in increments of 0.1 mg/day in the model. Additionally, we performed sensitivity analyses to test for the influence of using crude dietary fluoride intake (mg/day) as opposed to the dose of exposure, by generating a second model in which the exposure was a dose of fluoride exposure (mg/kg/day) and removing the weight’s summary measure as a covariate. Analyses were conducted using STATA v17.0 (StataCorp LP, College Station, TX, USA) and R v. 4.3.2 and the statistical significance was set at the 5% level. The packages mclogit23 (v0.9.6) and ordinal24 (v2023.12-4) were used to estimate the fixed and mixed cumulative logit link models; and ggplot225 (v3.4.4) to generate figures.

## 3. Results

### 3.1. Characteristics of the Participants, Dietary Fluoride Intake, and Dietary Covariates

Half of the participants in this study were females. During early childhood, about half of the participants were classified at the middle and lower end of the SES index. The participant’s mean duration of breastfeeding was 9.1 ± 6.5 months. At the adolescence visit, 47% of participants reported having started the use of fluoridated toothpaste between the age of 2–4 years. The distribution of participants was evenly distributed among the three ELEMENT recruitment cohorts (Table 1).

The recommended Adequate Intake levels (AI) for fluoride at 12 months, 2–3 years and >4 years are 0.5, 0.7 and 1 mg/day, respectively [8]. At all ages, participants had median dietary fluoride intakes (as estimated with the FFQ) above the recommended AI at all ages (*p* < 0.05, Wilcoxon Sign Rank test). The dietary intake of calcium ranged from 841 at year 1 to 1971 mg/day at age 5. Over the five early childhood visits, total energy intake estimated with the FFQ ranged from 1272 to 1971 Kcal/day, whereas children’s weight ranged from 9 to 18 kg (Table 2).

### 3.2. Distribution of Prevalence and Severity of Dental Fluorosis

**Figure 2:**
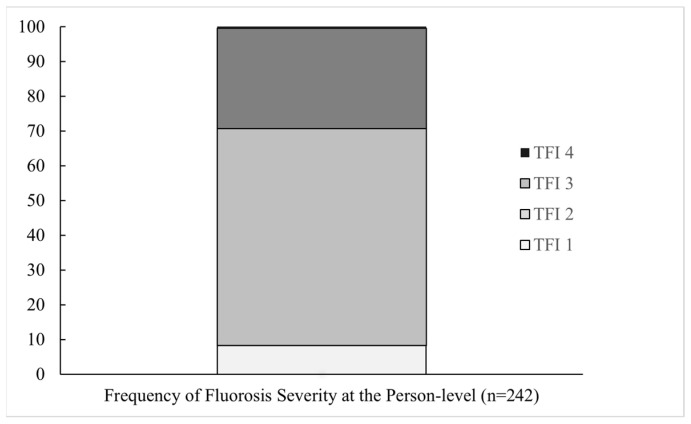
Frequency distribution of dental fluorosis severity assessed at adolescence (person level) in 242 participants of the Early Life Exposure to Environmental Toxicants in Mexico (ELEMENT) project by Thylstrup and Fejerskov Index (TFI) ordinal scores.

At the person level, almost all participants had some degree of dental fluorosis: only one person was classified as TFI 0 (0.05%); 9.2% were classified as TFI 1 and 65.5% as TFI 2 (very mild); 25.0% were classified as TFI 3 (mild), and only five (0.2%) were classified as TFI 4 (moderate). Therefore, this sample of adolescents is considered to have very mild to mild fluorosis (Figure 2).

**Figure 3:**
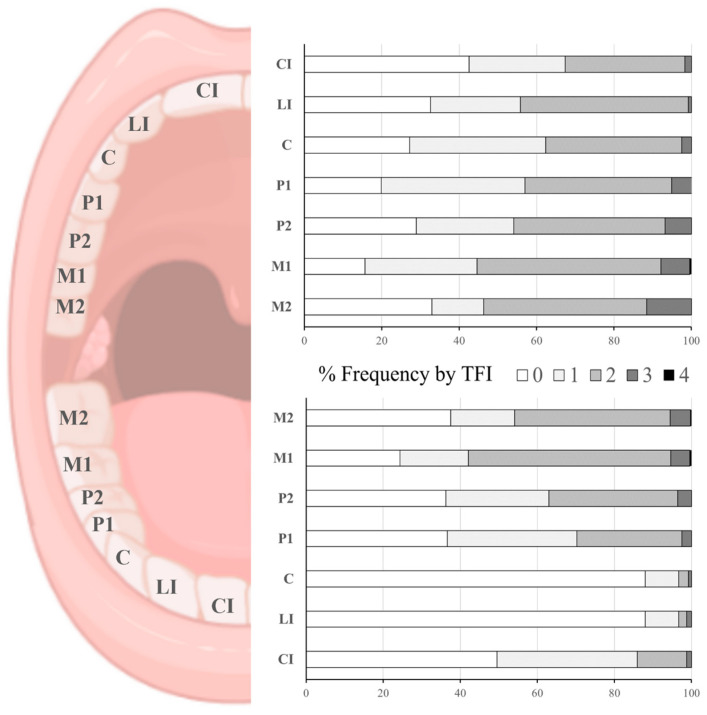
Frequency distribution of dental fluorosis occurrence and severity by tooth group, assessed with Thylstrup and Fejerskov index (TFI). Lack of dental fluorosis for a tooth group is scored as TFI = 0. Each participant (n = 242) was assigned a TFI score per tooth group. CI: central incisor, LI: lateral incisor C: canine, P1: 1st premolar, P2: 2nd premolar, M1: 1st molar, M2: 2nd molar.

At the tooth group level, lower central and lateral incisors had the lowest prevalence of fluorosis (TFI 0: 88.5% each). As expected from what was observed at the person level, very mild dental fluorosis (TFI 1–2) was the most prevalent in the assessed groups of teeth. Maxillary (upper) teeth had a higher occurrence of very mild fluorosis (TFI 1–2) compared to mandibular (lower) teeth. The occurrence of mild to moderate fluorosis (TFI 3–4) was low and observed mainly in posterior teeth (first premolars “P1, P2” and first molars “M1, M2”). Both maxillary and mandibular posterior exhibited the highest occurrence of fluorosis, from very mild to mild (TFI 1–3). Less than 0.2% of children had their molars scored as TFI 4 (Figure 3).

### 3.3. Association Between Levels of Dietary Fluoride Intake over the First Five Years of Life, and Change in TFI Scores in the Upper Central Incisors

**Figure 4:**
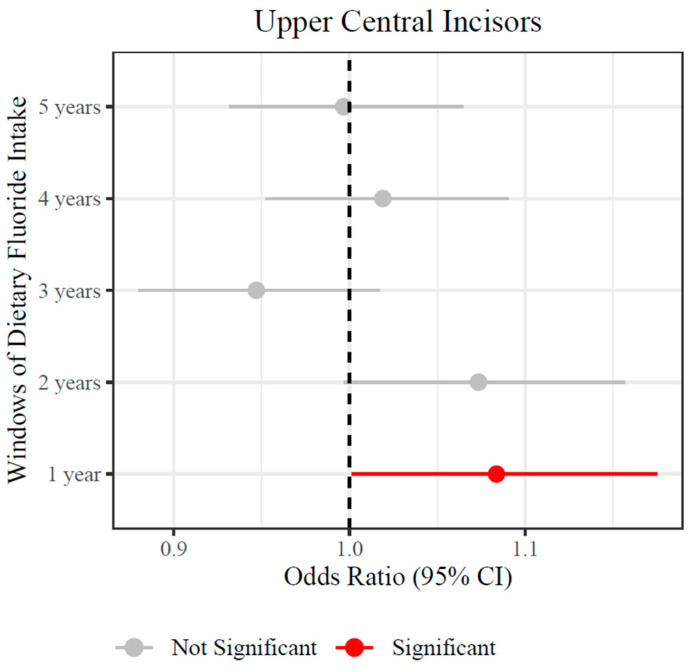
Forest plots for adjusted estimates [Odss Ratio, 95% Confidence Intervals] of the association between dietary fluoride intake (at windows 1–5 years) and Thylstrup and Fejerskov Index (TFI) ordinal scores in upper central incisors.

To identify the window(s) of dietary fluoride intake assessed annually over the first five years of life associated with increased odds of dental fluorosis in the upper central incisors, we used cumulative logit link regression models. To account for the confounding effect of the contribution of each individual window, estimated fluoride intake from all annual windows of exposure were included as predictors, whereas TFI scores in all tooth groups were the outcomes. We found that a 0.1 mg/day increase in dietary fluoride intake in the first year of life was associated with an average 1.08 increased odds of higher TFI score in the upper central incisors [95% CI: 1.00–1.17, *p*-value 0.05] (Figure 4 and Table S1). This means that holding constant all other windows of dietary intake (from 2 to 5 years), the dietary fluoride intake from solid foods at the first year of life was associated with increased odds of dental fluorosis in the upper central incisors. Interestingly, the estimated odds at year 2 were marginally significant [OR: 1.07; 95% CI: 1.00–1.16, *p*-value 0.06]. In sensitivity analyses where the predictors were doses of dietary fluoride intake per kilogram of weight (mg F/Kg/day), the results were consistent for the first year of life but not for the second (Table S2). All models were adjusted for important confounders such as duration of breastfeeding, dietary calcium intake, socioeconomic status, and age of initiation of fluoridated toothpaste use.

## 4. Discussion

In this sample of Mexican adolescents exposed to fluoridated salt, we found that their dietary fluoride intake during early childhood—estimated with an FFQ—exceeded the recommended adequate intake (AI) levels. While this higher intake can still offer protection against dental caries, it also increases the risk of dental fluorosis. H.T. Dean’s pioneering epidemiological research on fluoride and fluorosis suggested a prevalence threshold of 10% for mild fluorosis should be recognized as a public health concern [11]. As expected, we found mild dental fluorosis in all participants, indicating levels of exposure higher than what was considered acceptable in early fluorosis research.

Dental fluorosis has long served as the primary adverse health effect in fluoride dose–response analyses by the U.S. Environmental Protection Agency (EPA). However, a recent monograph by the U.S. National Toxicology Program (NTP) indicates that high levels of fluoride exposure may negatively impact cognition. The report emphasizes the need for additional research to understand the effects of fluoride at lower exposure levels. With data suggestive of a trend of increased dental fluorosis prevalence in the US [23], the focus on fluorosis is shifting from a primary adverse health effect to a biomarker of early fluoride exposure. In fact, a recent study used dental fluorosis as a secondary biomarker of fluoride exposure while investigating fluoride’s effects on Intelligence Quotient (IQ) [24].

The use of biomarkers in environmental exposure assessment involves the determination of associations between the biomarker and a measure of exposure [25]. Additionally, identifying critical windows of exposure improves risk assessment by identifying biologically relevant timeframes that should ideally coincide with the pathogenesis of the studied outcomes [26]. In this study, using longitudinal data from participants of the ELEMENT cohort, we found that controlling for each annual window of exposure, the first year of life was associated with the occurrence and severity of dental fluorosis in the permanent upper central incisors. Using annual assessments of dietary fluoride intake, we estimated that for each 0.1 mg of daily dietary intake of fluoride at age one (median intake of 0.56 mg/day in this sample), the odds of higher scores of dental fluorosis in the upper central incisors increase by 1.08. The only precedent on a longitudinal study in humans linking dietary intake of fluoride with dental fluorosis corresponds to reports from a cohort exposed to community levels of fluoride in water, “The Iowa Fluoride Study” (IFS) [15,16,27]. In the IFS, the occurrence of dental fluorosis in the upper central incisors and first molars was associated with total fluoride intake over the first three years of life [16], whereas for posterior permanent teeth, each window of exposure (from 2 to 8 years) but especially the sixth year, were associated with the occurrence of dental fluorosis [15]. In contrast to the IFS study, in our analyses, we were controlling for the effect of fluoride intake at all windows of exposure to identify the one with the most impact on the single group of teeth of interest (the UCIs), which was possible by fitting a cumulative logit link regression model using principal components of continuous confounders with repeated measures [28]. This approach significantly improves a cumulative logit model fit when there are predictors with repeated measures by avoiding the problem of multicollinearity and therefore improving the estimation of the model’s parameters.

In this study, the association of fluoride intake at the end of age one with increased odds of fluorosis cannot be attributed to differences in doses of dietary fluoride intake. Interestingly, crude dietary fluoride intake (mg/day) increased over the first five years, whereas fluoride intake doses by weight (mg/kg/day), were similar (Table 1). Historically, studies have assessed the relationship between dietary fluoride intake and dental fluorosis using the amount of fluoride consumed relative to body weight (mg/kg/day). However, in our primary models, we chose to use the total amount of fluoride consumed (mg/day) as the measure of exposure, while adjusting for body weight (kg) and total energy intake (kcal/day) as covariates in the model [29]. This decision was based on the fact that the organ system affected by fluoride in dental fluorosis is the developing enamel organ, which is independent of body size. Furthermore, the fluoride ion is not distributed to fatty tissues, and body composition is not expected to decrease local fluoride ion levels in the enamel organ [1]. Using estimates of intake by body weight would artificially decrease the exposure for heavier individuals, whose microcirculatory perfusion, and renal systems (which are the ones determining locally circulating fluoride levels) are not expected to be different from those of leaner individuals [30]. In fact, in our models, the association between fluoride intake at age two and fluorosis in the UCIs was lost when using fluoride intake doses by weight (Table S2). This finding could be due to the artificial decrease in fluoride intake caused by using the ratio of fluoride intake to the child’s weight as the exposure variable. Overall, our findings are consistent with the available evidence that associates the occurrence of dental fluorosis in the upper central incisors with fluoride exposure over the first two years of life [4] and validates its use as a biomarker in participants of the ELEMENT project.

The severity of fluorosis is determined by the local concentration of fluoride in the plasma that reaches the developing enamel organ [1], which is influenced by several physiological factors. For instance, bone—a physiological compartment—stores most of the circulating fluoride, especially during periods of bone growth and remodeling [2]. Bone accretion is minimal during the first year of life and increases progressively with the onset of walking and increased mechanical load on the skeleton [31,32]. Furthermore, fluoride is primarily eliminated by the kidneys, and infants have a slower rate of renal elimination of drugs due to a lower Glomerular Filtration Rate [33]. This suggests that the lack of fluoride uptake by bone in the first year may expose the developing enamel to higher local fluoride doses in the enamel organ compared to later years.

At 12 months of age, Mexican guidelines recommend that at least 60% of the daily energy intake should come from complementary foods, followed by human milk or infant formula [34]. Study participants were living in Mexico City during the length of the study, and an important source of dietary exposure captured by our FFQ is the intrinsic content of fluoride in solid foods. Although another limitation of this study is that we do not have data on infant formula intake, it is not expected to be the greatest source of dietary fluoride in a community with salt fluoridation [27]. Our reports of usual dietary intake at one year are likely underestimations that exclude fluoride intrinsic to the composition of infant formulas but include fluoride from cow’s milk, other beverages, and solid foods. To overcome this limitation, we included a variable on the duration of breastfeeding, as a higher intake of human milk is related to lower amounts of formula intake. A surprising finding was that the duration of breastfeeding was not associated with dental fluorosis and its estimates were positive (>1); however, this is not the first time that breastfeeding has been reported to potentially increase the odds of fluorosis [35]. There is evidence that short-term bone accretion is higher in formula-fed infants compared to breastfed infants in the first year of life, due to increased vitamin D and mineral content in formula compared to human milk [32]. The lower uptake of fluoride by the skeleton in breastfed infants could have resulted in higher local exposure to fluoride in the developing dental enamel over the first year. There is also the likelihood of unknown confounders being responsible for that association.

Another limitation of this study is that the validation of the FFQ used in the cohort was performed in women of childbearing age living in Mexico City. Therefore, there is a higher likelihood of measurement error, especially when applied at one year, when dietary intakes vary significantly within and between children. Furthermore, our study was not originally designed to study fluoride exposure, and the fluoride database used to calculate fluoride estimates was developed after the early childhood FFQ collection and did not include a comprehensive assessment of salt intake [21] which limits our ability to directly link fluorosis levels with fluoridated salt intake. The use of FFQs and food databases that do not exactly match the temporality of the data collection is a well-documented limitation inherent to the use of dietary assessment instruments [36]. To partially address this limitation, we were able to control for the effects of the recruitment cohort, which captures variation explained by the time of data collection. In addition, data collection of some of the covariates was performed at the adolescence visit (age of initiation of fluoridated toothpaste use) and is subject to recall bias. We were also unable to include other sources of fluoride intake such as toothpaste ingestion. Lastly, although we do not expect caregivers to add salt to children’s meals at the table (after cooking), we were unable to comprehensively capture this practice, leading to a potential risk of exposure misclassification. For future studies, we recommended conducting comprehensive dietary assessments and the use of UCIs as biomarkers of fluoride exposure.

Despite its limitations, this study has multiple strengths such as a longitudinal database (the second study of this kind), repeated measures of dietary fluoride intake, and the ability to control for important confounders and windows of exposure in the same model, such as dietary calcium intake, the age of initiation of fluoridated toothpaste use, socio economic status, weight, energy intake and cohort-specific effects. Additionally, we also followed rigorous training for the clinical assessment of fluorosis and included the age of the adolescent in our models as a confounding variable (as enamel abrasion due to routine toothbrushing, is associated with lower scores of dental fluorosis) [37]. Finally, although we included some of the most important covariates available to us, it is important to note that there is high interindividual variation in fluorosis severity scores. This variation is influenced by genetics [3], physiological factors, and other environmental factors that would hardly be accounted for by a single study [1,2].

## 5. Conclusions

In this sample of Mexican adolescents exposed to fluoridated salt, we found levels of dietary fluoride intake exceeding dietary recommendations and a high prevalence of mild dental fluorosis in permanent teeth. Furthermore, the dietary intake of fluoride reported at years 1 and 2 was associated with the occurrence and severity of dental fluorosis in the upper central incisors, which can serve as biomarkers of dietary fluoride exposure during the first two years of life in this cohort.

## Figures and Tables

**Table 1:** Characteristics of the study sample.

Variables	n = 432
Continuous	Mean (SD)
Age at childhood visits (months)	
1-yr	11.8 (0.4)
2-yrs	23.8 (0.4)
3-yrs	35.6 (2.3)
4-yrs	47.9 (0.8)
5-yrs	60.2 (1.0)
Age at adolescence visit (yrs)	14.6 (1.9)
Duration of breastfeeding (months)	9.1 (6.5)
Categorical	Freq (%)
Sex	
Female	214 (50)
Male	218 (50)
Socio Economic Status (SES)	
A/B, C+ (higher SES index)	96 (24)
C	129 (31)
D+	138 (29)
D/E	69 (16)
Age fluoridated toothpaste use started	
<2 yrs	113 (26)
2–4 yrs	205 (47)
4–6 yrs	87 (20)
>6 yrs	27 (6)
Recruitment Cohort	
2A	94 (22)
2B	102 (24)
3	236 (54)

**Table 2:** Summary statistics for longitudinal and dietary data. Values are means (SD) for ages at childhood visits, and medians (IQR) for the rest of the variables.

	Early Childhood Visits (n = 242)				
	1-yr	2-yrs	3-yrs	4-yrs	5-yrs
Dietary fluoride intake (mg/day)	0.56 (0.48)	0.91 (0.57)	1.11 (0.60)	1.14 (0.64)	1.12 (0.60)
Dose of dietary fluoride intake (mg/kg/day)	0.06 (0.05)	0.08 (0.06)	0.08 (0.04)	0.07 (0.04)	0.06 (0.04)
Dietary calcium intake (mg/day)	788 (979)	1339 (598)	1347 (498)	1381 (582)	1311 (561)
Total energy intake (Kcal/day)	1225 (717)	1736 (726)	1836 (738)	1946 (779)	1883 (723)
Weight (Kg)	9.05 (1.30)	11.60 (1.70)	13.78 (2.10)	15.80 (2.60)	18.00 (3.30)

## Data Availability

The data presented in this study are available on request from the cohort’s Principal Investigator following the process outlined at: https://sph.umich.edu/cehc/element/access_data.html (accessed on 13 February 2025).

## Supplementary Materials

The following supporting information can be downloaded at: https://www.mdpi.com/article/10.3390/ijerph22050689/s1, Table S1: Adjusted estimates [Odds Ratio, 95% Confidence Intervals] and *p*-values of the association between dietary fluoride intake (at windows 1–5 years) and ordinal Thylstrup & Fejerskov (TFI) in the upper central incisors. Dietary fluoride intake was fitted in the model in mg of fluoride intake per day (mg/day). Table S2: Adjusted estimates [Odds Ratio, 95% Confidence Intervals] and *p*-values of the association between dietary fluoride intake (at windows 1–5 years) and ordinal Thylstrup & Fejerskov (TFI) in the upper central incisors. Dietary fluoride intake was fitted in the model in dose of intake per kg of body weight (mg/kg/day).
