# Supplementary material for: Dietary Fluoride Exposure During Early Childhood and Its Association with Dental Fluorosis in a Sample of Mexican Adolescents
Source: Int J Environ Res Public Health. 2025 Apr 26;22(5):689. doi: 10.3390/ijerph22050689 (PMC12111587; doi:10.3390/ijerph22050689)
Supplement: Supplementary file 1 [file ijerph-22-00689-s001.zip › ijerph-3556100-supplementary.pdf]

**Table S1.** Adjusted estimates [Odds Ratio, 95% Confidence Intervals] and p-values of the association between dietary fluoride intake (at windows 1 - 5 years) and ordinal Thylstrup & Fejerskov (TFI) in the upper central incisors. Dietary fluoride intake was fitted in the model in mg of fluoride intake per day (mg/day).

| n=242 | Upper Central Incisors | | |
|---|---|---|---|
| | **OR** | **95% CI** | ***p-value*** |
| Dietary Fluoride Intake (mg/day) | | | |
| 1 year | 1.95 | 1.00 - 1.17 | *0.05* |
| 2 years | 1.84 | 1.00 - 1.16 | *0.06* |
| 3 years | 0.49 | 0.88- 1.02 | *0.14* |
| 4 years | 1.28 | 0.95 - 1.09 | *0.66* |
| 5 years | 0.78 | 0.93 - 1.07 | *0.92* |

**Table S2.** Adjusted estimates [Odds Ratio, 95% Confidence Intervals] and p-values of the association between dietary fluoride intake (at windows 1 - 5 years) and ordinal Thylstrup & Fejerskov (TFI) in the upper central incisors. Dietary fluoride intake was fitted in the model in dose of intake per kg of body weight (mg/kg/day).

| n=242 | Upper Central Incisors | | |
|---|---|---|---|
| | **OR** | **95% CI** | ***p-value*** |
| Dietary Fluoride Intake (mg/Kg/day) | | | |
| 1 year | 1.06 | 1.00 - 4.06 | *0.05* |
| 2 years | 1.02 | 0.84 - 4.06 | *0.13* |
| 3 years | 0.98 | 0.18- 1.25 | *0.16* |
| 4 years | 1.03 | 0.48 - 3.32 | *0.69* |
| 5 years | 0.98 | 0.25 - 2.44 | *0.61* |
